# Acidic microenvironment triggered *in situ* assembly of activatable three-arm aptamer nanoclaw for contrast-enhanced imaging and tumor growth inhibition *in vivo*

**DOI:** 10.7150/thno.72028

**Published:** 2022-04-24

**Authors:** Jin Huang, Yuchen Wu, Hui He, Wenjie Ma, Jianbo Liu, Hong Cheng, Huanhuan Sun, Xiaoxiao He, Kemin Wang

**Affiliations:** State Key Laboratory of Chemo/Biosensing and Chemometrics, College of Biology, College of Chemistry and Chemical Engineering, Hunan University, Key Laboratory for Bio-Nanotechnology and Molecular Engineering of Hunan Province, Changsha 410082, China.

**Keywords:** *in situ assembly of multivalent nanodevice*, * in situ* enhanced binding affinity, acidic tumor microenvironment, contrast-enhanced tumor imaging, tumor growth inhibition

## Abstract

Rationale: Static assembled multivalent DNA nanotheranostics system have encountered some bottleneck problems in cancer imaging and therapy, such as poor penetration and high immunogenicity. Herein, we proposed an acidic tumor microenvironment triggered assembly of activatable multivalent nanodevice, called “three-arm aptamer nanoclaw” (TA-aptNC), assembled from three pH-responsive aptamer-decorated DNA monomers (pH-aptDMs) to facilitate their functions of imaging and therapy.

Methods: The activated TA-aptNC was constructed by acidic microenvironment triggered *in situ* assembly of three pH-aptDMs. Designer pH-aptDM was established based on the combination of a pH-responsive i-motif switch and an assembly module with a cell membrane anchoring aptamer ligand. Acidic microenvironment-triggered the assembly of the TA-aptNC was characterized by electrophoresis and atomic force microscopic (AFM). The binding affinity and stability of the TA-aptNC, comparing the monovalent pH-aptDM, were studied via the flow cytometry and nuclease resistance assays. Acidic microenvironment-activated contrast-enhanced tumor imaging and significantly antitumor efficiency were evaluated *in vitro* and *in vivo*.

**Results**: At physiological pH environment, the pH-aptDMs with excellent tissue permeability exited as inactivated and monodispersed small monomer. When encountering acidic microenvironment at the tumor site, pH-responsive i-motif switch liberated from the pH-aptDMs, and the three unconstrained DNA modules (DM_1_, DM_2_ and DM_3_) subsequently assembled* in situ* into the TA-aptNC. Compared with monovalent pH-aptDMs, the spontaneously formed activatable TA-aptNC afforded 2-fold enhanced binding ability via the multivalent effect, which further facilitated the selective tumor cell uptake capability, thus enabling a contrast-enhanced tumor imaging and significantly antitumor efficiency* in vivo* without systemic toxicity.

**Conclusions**: The proposed strategy offers valuable insight into excavating an endogenous stimuli-triggered assembly of multivalent nanodevice for accurate diagnosis and efficient tumor therapy.

## Introduction

Aptamers termed as 'chemical antibodies', are short oligonucleotide sequences with a folded three-dimensional structure, which can selectively bind to specific small molecules, proteins, or the whole cells 1,2. Owing to the unique advantages of high selectivity, specificity, easy synthesis and modification, aptamers promise an emerging molecule tool for applications in tumor detection 3,4, imaging 5-7, and therapy 8-10. As powerful recognition ligands, aptamers have been extensively explored to be labelled with other units (isotopic indicators, radionuclides, fluorescent dyes, and drug molecules) or assemble into aptamer-decorated DNA nanostructures, generating monovalent aptamer probes 9,11. Despite monovalent aptamer probes showing the potential applications in disease diagnosis and therapy, the unsatisfactory affinity and poor stability in the complex physiological environment inevitably restrict their widespread applications *in vivo* 6,12.

To meet these requirements, multivalent aptamer probes, by integrating multiple aptamer units into one nanomaterial or DNA nanostructure, have been employed to enhance the binding affinity via the multivalent effect. It has been shown the multivalent aptamer probes strategy increased local ligand density or synergistic interaction 13-15. In particular, benefiting from the great progress of DNA nanotechnology, series of DNA nanostructures including DNA tetrahedron 10,16,17, DNA nanowire 18, DNA nanohydrogel 19,20, RCA-based DNA nanostructure 21,22, etc., have been exploited and used as scaffolds to incorporate multivalent aptamers. Despite the progress has made, the preassembled DNA nanostructures, also known as “static assembled multivalent nanodevices”, often possess the characteristic of large size, which makes them difficult to penetrate into the entire surface-to-core tumor tissues and prone to be cleared by the reticuloendothelial system* in vivo* 23,24. Besides, the multiple aptamers are embedded in various sites of DNA scaffold by an imprecise controlled manner, which inevitably results in higher steric hindrance, thus affording undesired improvement of binding affinity 21,25,26.

Recently, the concept of *in situ* assembly in specific site has been proposed for cancer imaging and therapy. *In situ* self-assembly is a process that exogenous small monomers integrating stimulus-responsive primitives and assembly units self-assemble into specific architecture by the stimulation of the pathological abnormalities 27. Endogenous stimulus-triggered *in situ* assembly strategy shows some prominent merits: first, they can not only transform the morphology of functional nanostructures, but also facilitate their functions of imaging or therapy 28-30. Second, this strategy imitates naturally occurring process at physiological environment, which is essential to understand the mechanism of disease occurrence and guide the therapy precisely 31. Currently, the distinctive tumor microenvironment (TME) of solid tumors, such as acidic extracellular pH, overexpressed proteins/enzymes and hypoxia 32,33, as potential endogenous triggers of* in situ* assembly methods provides an alternative opportunity to spatiotemporally manipulate biological behaviors at special site. Especially, acidic tumor microenvironment as ubiquitous stimulus induces the conformation transition of pH-responsive units (i-motif, triplex DNA, pH-sensitive amphiphilic polymers and so on) for realizing precise assembly/disassembly 34-36, promising potential applications in construction of *in situ* assembled multivalent nanodevice for modulation of binding affinity and cellular internalization behavior at tumor site. Thus motivated, our group first reported extracellular pH-powered *in situ* assembly of multivalent DNA assemblies by the crosslink of aptamer functional DNA monomer on the cell surface, which achieved enhanced binding affinity and drugs delivery 37. Nevertheless, this strategy employed an “always-on” mode for cancer diagnosis and therapeutic, leading to a long diagnosis time and poor imaging contrast. Subsequently, we continuously developed acid-responsive assembly of multivalent nanotheranostics systems based on activatable strategy and successfully used to realize contrast-enhanced imaging of cancer cells. One is acid-activated reconfiguration of DNAzyme-based multivalent theranostics nanodevice for cancer cells imaging and gene therapy 38. The other is pH-driven *in situ* constructed of activatable multivalent theranostics platform for intracellular TK1 mRNA activated cancer cells imaging and killing 39. Essentially, these encouraging attempts at stimuli-responsive assembly of activatable multivalent nanotheranostics system demonstrated their superiority of contrast-enhanced imaging, enhanced cell killing and lower normal tissue toxicity. However, both reports are inevitably limited by the undefined aptamer density, imprecise controlled spatial orientation and physical dimension, thereby leading to the undesirable improvement of binding. Additionally, these above-mentioned *in situ* assemblies of multivalent nanotheranostics systems were initiated in a simulated solution. Therefore, it is desirable to excogitate endogenous stimuli-responsive *in situ* assembly strategy to construct precisely controlled activatable multivalent nanodevice *in vivo*.

**Scheme 1:**
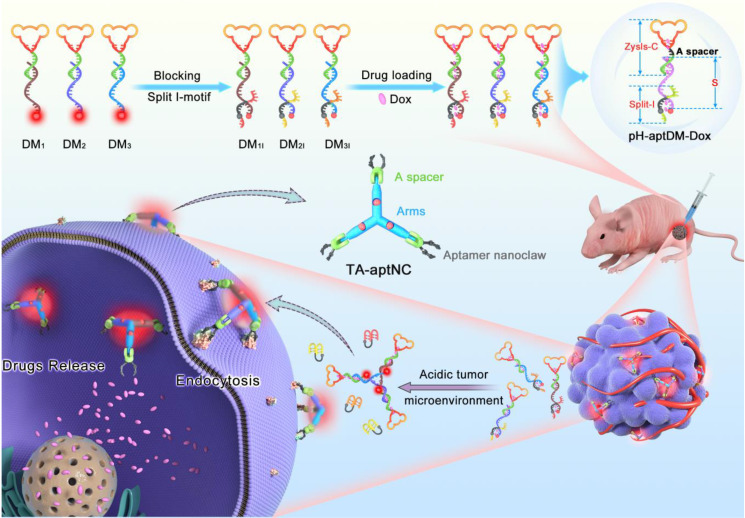
** Schematic illustration of acidic tumor microenvironment-triggered *in situ* assembly of activatable multivalent nanodevice (TA-aptNC) for contrast-enhanced imaging and antitumor growth.** In the free state, three pH-responsive aptamer-decorated DNA monomer (pH-aptDMs, including DM_1I_, DM_2I_ and DM_3I_) exited as monodispersed small monomer with quenched fluorescence, showing excellent tissue permeability. When the mixture of three pH-aptDMs was administered into SMMC-7721 tumor-bearing nude mice via an intratumoral injection, the mixture responses to the acidic tumor microenvironment (pH 6.2-6.9), leading to the assembly of activatable TA-aptNC. The TA-aptNC with *in situ* improved binding affinity specifically targeted tumor cell, which further facilitates rapid drug delivery, thereby showing a contrast-enhanced* in vivo* tumor imaging and a significant antitumor growth efficiency.

For cancer diagnosis and therapy, there is still a blank area to improve the imaging contrast and therapy using an *in situ* assembly multivalent nanotheranostics at tumor site. Based on our previous design, we first proposed an acidic tumor microenvironment-powered autonomous *in situ* assembly of activatable multivalent strategy by designing three pH-responsive and aptamer-decorated DNA monomers (pH-aptDMs). The resultant activated multivalent nanodevice, called “three-arm aptamer nanoclaw” (TA-aptNC), possessed the unique superiorities of the precise controlled spatial orientation, defined ligand density and structure size, which is crucial for the improvement of binding avidity and cellular internalization efficiency. As illustrated in **Scheme 1**, three DNA modules (DM_1_, DM_2_ or DM_3_) were designed to be partly complementary to each other. Each of them has a sticky-end that was respectively locked by the split i-motif sequence (I_1_, I_2_ or I_3_), generating pH-responsive and aptamer-decorated DNA monomers (pH-aptDMs, including DM_1I_, DM_2I_ or DM_3I_). Doxorubicin, a model anticancer drug, embed into the abundant CG base pairs of pH-aptDMs. At physiological condition (pH 7.2-7.4), three pH-aptDMs existed as monodispersed structure, showing excellent tissue permeability and inactivated fluorescence due to the fluorophore in close proximity to the quencher. When encountering the acidic tumor microenvironment (pH 6.2-6.9), the protonated split i-motif (I_1_, I_2_ and I_3_) could form quadruplex structures and then liberate from DNA monomers, thus resulting in fluorescence recovery and partial drugs release. Three DNA modules with the unconstrained sticky ends would then be assembled with each other to form the TA-aptNC via sticky-end hybridization at tumor site. Three aptamers respectively positioned on the three outstretched arms of TA-aptNC, as “recognition claws”, were able to grasp the receptor on the cell membrane. The resultant multivalent effect could accelerate the internalization of the TA-aptNCs into target tumor cell via receptor-mediated internalization pathways, thus improving the drug delivery and greatly inhibiting tumor growth *in vivo*.

As far as we know, this is the first report of endogenous stimulus-powered *in situ* assembly of precisely controlled multivalent aptamer nanodevice for facilitating their imaging and therapy functions *in vivo*. Irrespective of the introduction of any exogenous additives, the TA-aptNC-based *in situ* assembly system not only allowed acidic tumor microenvironment-activated contrast-enhanced *in vivo* imaging and selective cytotoxicity to target tumor cells, but also afforded significantly antitumor efficiency *in vivo*. As a modularly molecular design concept, targeting module, stimuli- responsive module and assembling module were intelligently integrated to form a multifunctional pH-aptNC. Furthermore, the striking features of DNA-based multivalent system are biocompatible, biodegradable and no self-toxicity.

## Materials and Methods

### Preparation of three monomer

The pH-aptDMs (DM_1I_, DM_2I_ and DM_3I_) were prepared via an annealing protocol. In the experiment, the DM_1I_ monomer was assembled from three oligonucleotide strands (ZYsls-C, S_1_ and I_1_) with a molar ratio of 1:1:1.5 in 10 mM PBS (pH 7.4). Then, the mixture was annealed by using enzyme-free self-assembled procedure: 95 °C for 5 min, 70 °C for 10 min, 60 °C for 10 min, 50 °C for 15 min, 40 °C for 20 min, 30 °C for 30 min and 20 °C for 30 min. Similarly, the self-assembly of DM_2I_ (ZYsls-C, S_2_ and I_2_), DM_3I_ (ZYsls-C, S_3_ and I_3_) and control probes were synthesized by following the same methods. A slightly excessive blocking sequence (split i-motif, including I_1_, I_2_ and I_3_) was respectively added to sufficiently locking the sticky ends of pH-aptDMs. The formation of pH-aptDMs was finally verified by native Gel Electrophoresis.

### Gel electrophoresis analysis

All probes were dissolved in buffer (1 μM), respectively. The stepwise assembly of DM_1I_, DM_2I_ and DM_3I_ in PBS (pH 7.4) was estimated by 3% agarose gel electrophoresis. To characterization of acidic pH-triggered* in situ* assembly of three pH-aptDMs, the mixture of DM_1I_, DM_2I_ and DM_3I_ (1 μM) were incubated in PBS (pH 7.4 or 6.3) at 37 °C for 1 h. Gel electrophoresis analysis were performed in 1× TBE buffer at 4 °C for 1 h under a constant potential of 100 V. For imaging, the gel was then stained in SYBR Gold for 30 min, finally photographed by an Azure C600 Imaging Biosystems (CA).

### Flow cytometry assays

To evaluate the acidic pH-powered *in situ* assembly multivalent system for fluorescent labeling of target cells, equal DM_1I_, DM_2I_ and DM_3I_ (15 nM) were co-incubated with 1.5 × 10^5^ target tumor cells in binding buffer (pH 7.4 or 6.3) at 37 °C for 1 h. Subsequently, it was determined by a Gallios cytometer (Beckman Coulter, USA) by counting 10,000 events.

To evaluating the binding capacity of TA-aptNC-based *in situ* assembly strategy at cellular level, different probes were reasonably designed, including TA-CaptNC (the mixture of CDM_1I_, CDM_2I_ and CDM_3I_, aptamer sequence replaced by arbitrary sequences), pH-C (the mixture of DM_1C_, DM_2C_ and DM_3C_, split i-motif sequence substituted by pH-insensitive sequence), pH-aptDM (the corresponding monovalent probe) and TA-aptNC (the mixture of DM_1I_, DM_2I_ and DM_3I_). These probes were respectively treated with the target cells and then determined by a Gallios cytometer (Beckman Coulter, USA).

### Laser Scanning Confocal Microscopy imaging

To investigate the acidic tumor microenvironment-triggered fluorescent labeling of TA-aptNC-based strategy, cells were firstly plated in confocal dish and incubated at 37 °C in 5% CO_2_ for 24 h, then washed three times using the binding buffer before incubation. After SMMC-7721 cells treated with different composition (pH-C, pH-aptDM, TA-aptNC) in binding buffer at pH 6.3 or 7.4 for 1 h in the dark. Then, the cells were washed with PBS 3 times and stained with Hoechst 33342 before observation. Samples were monitored using microscope (Nikon, Japan) with a 100× objective. Note that the concentrations of these probes were regulated to have the same amount of Cy5 and aptamer.

To investigate the specifical binding of *in situ* assembled TA-aptNC to target SMMC-7721 cells, the mixture of three pH-aptDMs were co-incubated with different cells (target SMMC-7721, HeLa, HepG2, MCF-7 and L02 cells) in different pH at 37 °C for 1 h. After incubation, all samples were washed 2 times and then resuspended in PBS for microscopic observation.

### Intracellular delivery of Dox

The localization of Dox and Cy5-TA-aptNC was studied by incubating SMMC-7721 cells with different compositions. Cy5-pH-aptDM or Cy5-TA-aptNC and Dox (mole rations of 1:5) were firstly mixed to prepare Cy5-pH-aptDM-Dox and Cy5-TA-aptNC-Dox as described above. subsequently, different compositions, including free Dox, Cy5-TA-aptNC-Dox (the mixture of Cy5- DM_1I_-Dox, Cy5- DM_2I_-Dox and Cy5- DM_3I_-Dox), Cy5-pH-aptDM-Dox, Cy5-TA-aptNC (the mixture of Cy5- DM_1I_, Cy5- DM_2I_ and Cy5- DM_3I_), or Cy5-pH-aptDM, were respectively incubated with SMMC-7721 cells at 37 °C in CO_2_ incubator for 1 h, then removing the medium. All samples were washed twice with PBS. Hoechst 33342 was added for specific staining of the nucleus for 10 min. The samples were washed with PBS twice, and then resuspended in binding buffer (1000 µL) for cellular fluorescent images by Nikon A1 confocal laser scanning microscopy with a 100× objective. Fluorescent imaging of Hoechst 33342 was detected using 405/430 nm excitation/emission filters. Dox was detected using 488/505 nm. Cy5 was detected using 633/660 nm.

### Cytotoxicity assay

MTS assay was employed to evaluate cell viability. 0.2 mL SMMC-7721 cells were seeded in 96-well culture plates at a density of 1 × 10^4^ cells per well for 2 h. Cells were then treated with the corresponding probes at different concentrations for 3 h. After 80% of the supernatant medium removed, 0.2 mL RPMI 1640 medium (12% FBS) was added for further cell growth until 48 h. Last, 20 μL of MTS reagent diluted in 100 μL of cell culture medium was added to incubate with the cells for 2 h at 37 °C. Finally, the absorbance of 490 nm was obtained by using a microplate reader. (Bio Tek Instrument, Inc.). Cell viability was calculated according to manufacturer's description.

### *In vivo* fluorescence imaging

All animal studies were performed in compliance with institutional animal use and care regulations, according to protocol No. SYXK (Xiang) 2018-0006, approved by the Laboratory Animal Center of Hunan Province. Male BALB/c nude mice were purchased from Hunan SJA Laboratory Animal Co., Ltd. All mice used in this experiment are 4-6 weeks old at the start of each experiment and weighed 20-25 g. To establish the mouse xenograft tumor models, male nude mice were subcutaneously injected with 1 × 10^6^ SMMC-7721 cells on the right back of the mouse. The SMMC-7721-implanted mice were then allowed to grow for 3-4 week until the tumor reached 1 cm in diameter. The mice were randomized into 3 groups (five mice each group) and administrated with 1.5 μΜ of pH-C, pH-aptDM and TA-aptNC into the tumor tissue by intratumoral injection and the corresponding normal muscle tissues. The real-time fluorescence images were monitored by an IVIS Lumina II *in vivo* imaging system (Caliper Life Science, U.S.A.) at 0, 1, 5, 15, 30, 45 and 60 min. The mice were anesthetized with anesthetic before imaging.

### Tissue slice imaging

For tissue slice imaging, when the mice were deeply anesthetized, SMMC-7721 tumor-bearing mice were firstly sacrificed. The fresh tumor tissues and normal muscle tissues were excised, immediately embedded in OCT and frozen at -80 °C for several minutes. The frozen tissues were then cut into 8 μm slices using a frozen tissue slicer of CM1850 (Leica, Germany). Subsequently, the frozen tissue slices were quickly adsorbed on glass slides for the assessment of the acidic tumor microenvironment-triggered *in situ* assembly of three pH-aptDMs into TA-aptNC. 1.5 μM of the corresponding probes were respectively added to the central area of the tissue section. After incubation for 1 h at 37 °C, the slicer fluorescent images were collected on a Nikon confocal microscope with a 60× oil immersion objective.

### *In vivo* tumor growth inhibition

Male BALB/c mice with SMMC-7721 tumor-bearing xenograft, when the tumor volume reached 100 mm^3^, were randomly allocated into three groups (five mice each group) and administered with PBS, TA-aptNC (the mixture of DM_1I_, DM_2I_ and DM_3I_) and TA-aptNC-Dox (the mixture of DM_1I_-Dox, DM_2I_-Dox and DM_3I_-Dox) by intratumoral injection. Dox dose of 0.5 mg/kg-1 were injected every other day for a total of six injections. The same amount of PBS and the mixture of DM_1I_, DM_2I_ and DM_3I_ were treated with the control group, respectively. The changes of body weight and tumor volumes were monitored and recorded every other day for 14 days. The tumor volume was obtained as the following formula: tumor volume = Length × Width^2^/2 using a caliper. At the end of the relevant timeline (day 14), the animals were sacrificed by cervical dislocation under general anesthesia. The tumors were harvested for further histological analysis.

## Results and Discussion

### Fabrication and characterization of the pH-responsiveness of TA-aptNC-based *in situ* assembly system

**Figure 1:**
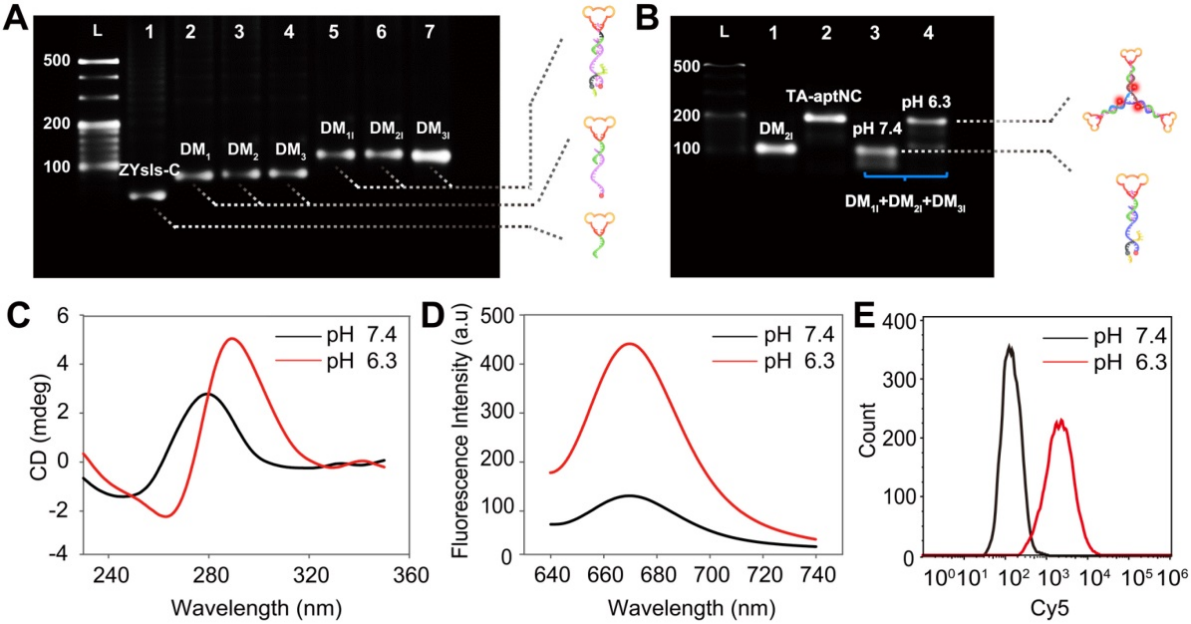
** Characterization of pH-triggered *in situ* construction of TA-aptNC. (A)** 3% agarose gel electrophoresis analysis of the stepwise assembly of DNA monomers. Lane L, 20 bp DNA ladder; Lane 1, ZYsls-C; Lane 2, ZYsls-C + S_1_ (DM_1_); Lane 3, ZYsls-C + S_2_ (DM_2_); Lane 4, ZYsls-C + S_3_ (DM_3_); Lane 5, ZYsls-C + S_1_ + I_1_ (DM_1I_); Lane 6, ZYsls-C + S_2_ + I_2_ (DM_2I_); Lane 7, ZYsls-C + S_3_ + I_3_ (DM_3I_). **(B)** 3% agarose gel electrophoresis verification of the formation of TA-aptNC. Lane L, 20 bp DNA ladder; Lane 1, DM_2I_; Lane 2, TA-aptNC (annealing assembly); Lane 3, DM_1I_ + DM_2I_ + DM_3I_ at pH 7.4; Lane 4, DM_1I_ + DM_2I_ + DM_3I_ at pH 6.3. The CD spectra **(C)** and fluorescence spectra **(D)** characterization of the mixture of three pH**-**aptDMs in the simulated buffer. Flow cytometry analysis of the target SMMC-7721 cells **(E)** after treated with the mixture under different pH values.

In this strategy, the aptamer ZYsls, selected by our group, was used as model function domain against human liver cancer SMMC-7721 cells. To ensure that the whole system can operate as expected, the stepwise assembly of three pH-aptDMs was characterized by 3% agarose gel electrophoresis, showing an obvious band migration and high yield **(Figure 1A)**. In this strategy, acidic pH as a trigger could turn on the pH-switch integrated into three pH-aptDMs, and then power unblocked DNA modules assembled into activated TA-aptNC. The feasibility of our strategy was explored by electrophoresis assays **(Figure 1B)**. Lanes 1 and 2 represented pH-aptDM (DM_2I_) and TA-aptNC formed by annealing assembly, respectively. Due to the excellent stability of split i-motif sequences at pH 7.4, the mixture of three pH-aptDMs failed to assemble into TA-aptNC, resulting in unnoticeable band movement in lane 3. In comparison, there existed two bands with different gradients representing the activated TA-aptNC and the residual pH-aptDMs, demonstrating successful activation of the assembly of TA-aptNC at pH 6.3. The pH-triggered formation of the TA-aptNC was then characterized by atomic force microscopic (AFM). As expected, a series of three arms-like DNA nanostructures with an average size of ~15 × 2 nm were observed at pH 6.3, indicating pH-triggered the successful assembly of three pH-aptDMs into TA-aptNC (**Figure S1**). The pH-responsiveness of pH-aptDM was further characterized by circular dichroism (CD) spectroscopy. Compared with free ZYsls-C **(Figure S2A)**, the pH-aptDM monomer and the mixture of three pH-aptDMs, similar to the free split i-motif, exhibited distinct red shift of both positive and negative bands at pH 6.3 owing to base stacking and polynucleotide helicity of unstructured single-stranded DNA **(Figure 1C, S2B-C)** 40,41. The results indicated that acidic pH-driven conformational switch went from the random coil to intact i-motif successfully.

Furthermore, the fluorescence spectra analysis also revealed the pH-response performance of the proposed strategy by employing the pH-insensitive fluorophore/quencher pair (Cy5/BHQ2) **(Figure S3)**. We first optimized the assembly ratio of BHQ2-I to Cy5-S in pH-aptDM **(Figure S4)**. On the basis of the above optimized results, the pH-responsiveness of pH-aptDM was monitored. As shown in **Figure 1D**, the mixture of three pH-aptDMs could exist as monodispersed small monomer at pH 7.4 and gave a low background fluorescence signal. When adjusting the pH to 6.3, BHQ2-I disassembled from Cy5-S, resulting in a notable activated fluorescence increase. Three monomers also displayed a similar phenomenon **(Figure S5)**, indicating the excellent pH-response performance of pH-aptDMs. Inspired by the results from cell-free system, flow cytometric analyses of target SMMC-7721 cells **(Figure 1E)** were also employed to investigate the feasibility of pH-triggered *in situ* assembly of multivalent nanodevice. The analyses indicated that the *in situ* assembled TA-aptNC exhibited an activatable fluorescence signal response to target SMMC-7721 cells at pH 6.3. Together, these results showed that split i-motif unit integrated into the pH-aptDM as a pH-responsive switch could manipulate the assembly of DNA modules into multivalent nanodevice in response to acidic pH.

### Evaluation of the binding performance of the TA-aptNC-based *in situ* assembly system

**Figure 2:**
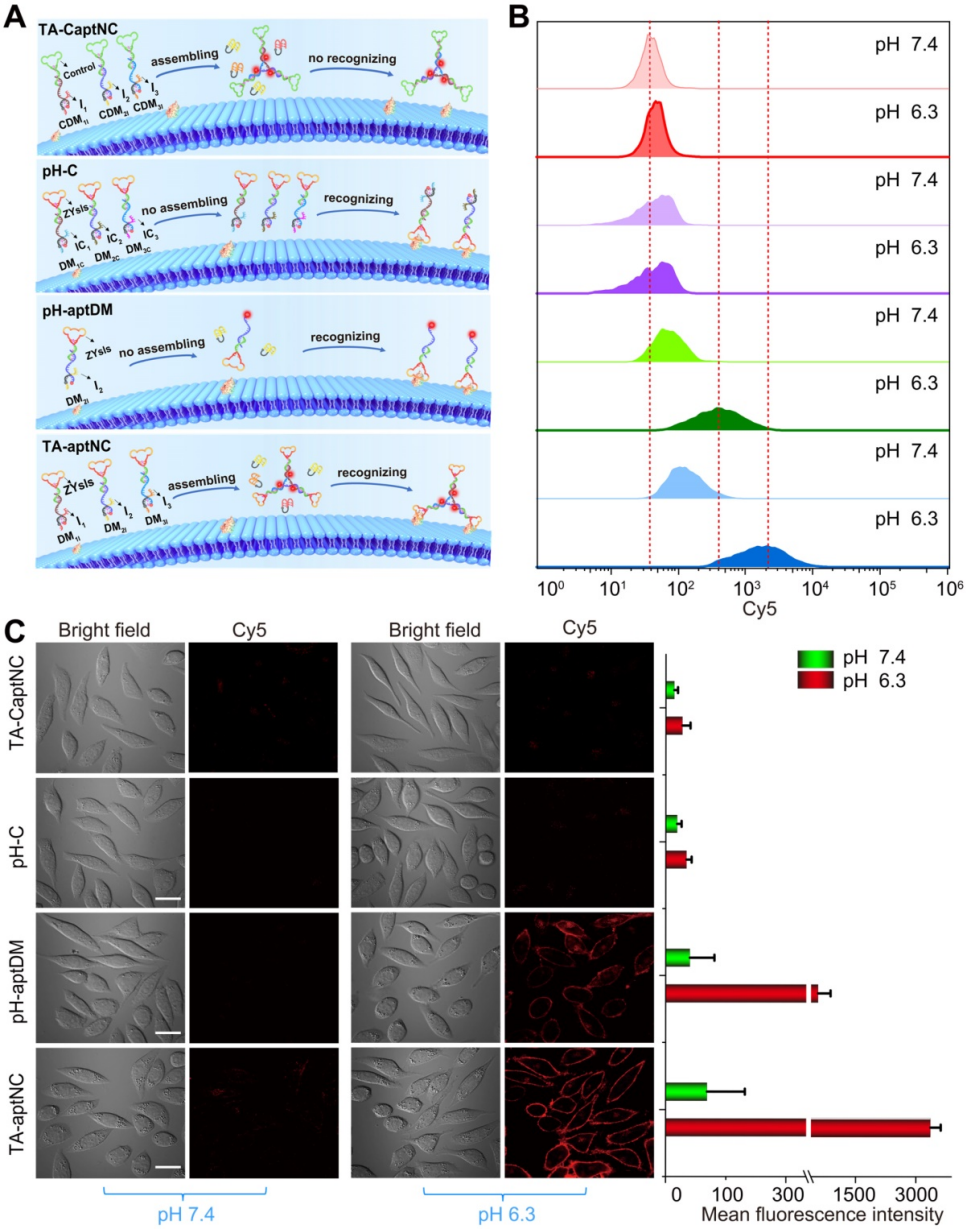
** Binding behavior investigation of pH-triggered *in situ* assembly of multivalent nanodevice. (A)** Schematic diagram of pH-triggered the *in situ* assembly of multivalent nanodevice and binding behavior of various compositions at pH 6.3. Flow cytometry analysis **(B)** and confocal microscopy assays **(C)** of SMMC-7721 cells after incubation with different compositions in binding buffer with different pH at 37 ^o^C for 1 h. The corresponding histogram of the four compositions show the mean fluorescence intensity in the images (C) (scale bar: 20 μm). Data are represented as means ± SD (n = 3).

The binding capacity of TA-aptNC-based *in situ* assembly strategy was investigated at cellular level. To make a reasonable comparison, a variety of control probes were reasonably designed, including TA-CaptNC (the mixture of CDM_1I_, CDM_2I_ and CDM_3I_, aptamer sequence replaced by arbitrary sequences), pH-C (the mixture of DM_1C_, DM_2C_ and DM_3C_, split i-motif sequence substituted by pH-insensitive sequence) and pH-aptDM (the corresponding monovalent probe). Note that these probes needed to be adjusted to possess the same concentration of targeting ligand ZYsls and Cy5. After treated with different compositions at pH 6.3 **(Figure 2A)**, the flow cytometric analyses demonstrated that the* in situ* formed TA-aptNC acquired high fluorescence labelling of target cells **(Figure S6)**, exhibiting an excellent SNR that was ~30 times of control compositions (TA-CaptNC and pH-C). While target cells treated with four compositions at different pH, the pH-aptDM and the mixture of three monomers (TA-aptNC group) showed discernible signal in response to target SMMC-7721 cells at pH 6.3 **(Figure 2B and S7).** The latter established outstanding SBR that was ~8 times of pH-C, which was ~2 times higher than that of the former. In contrast, despite the mixture of CDM_1I_, CDM_2I_ and CDM_3I_ could be assembled into TA-CaptNC with activated signal at pH 6.3, the poor affinity prevented it from targeting SMMC-7721 cells. As to pH-C, the acid-insensitive performance made it difficult to be activated at pH 6.3, thus generating undistinguished fluorescence labelling. Meanwhile, the confocal laser scanning microscopy images also revealed the binding advantage of *in situ* formed TA-aptNC to target cell **(Figure 2C)**. An ignorable fluorescence was observed on the membrane of cells under neutral pH, which is due to the probes failing to be activated. When adjusting the pH to 6.3, the mixture of three monomers incubated with SMMC-7721 cells for 1 h displayed twinkle halos around cells, much more than that of the pH-aptDM under the same conditions. The corresponding mean fluorescence intensity of Cy5 labelled SMMC-7721 cells once again highlighted the excellent binding capability of the TA-aptNC-based *in situ* assembly strategy. Together, these results strongly validate that acidic pH-activated *in situ* assembly multivalent system could greatly enhance image contrasts.

**Figure 3:**
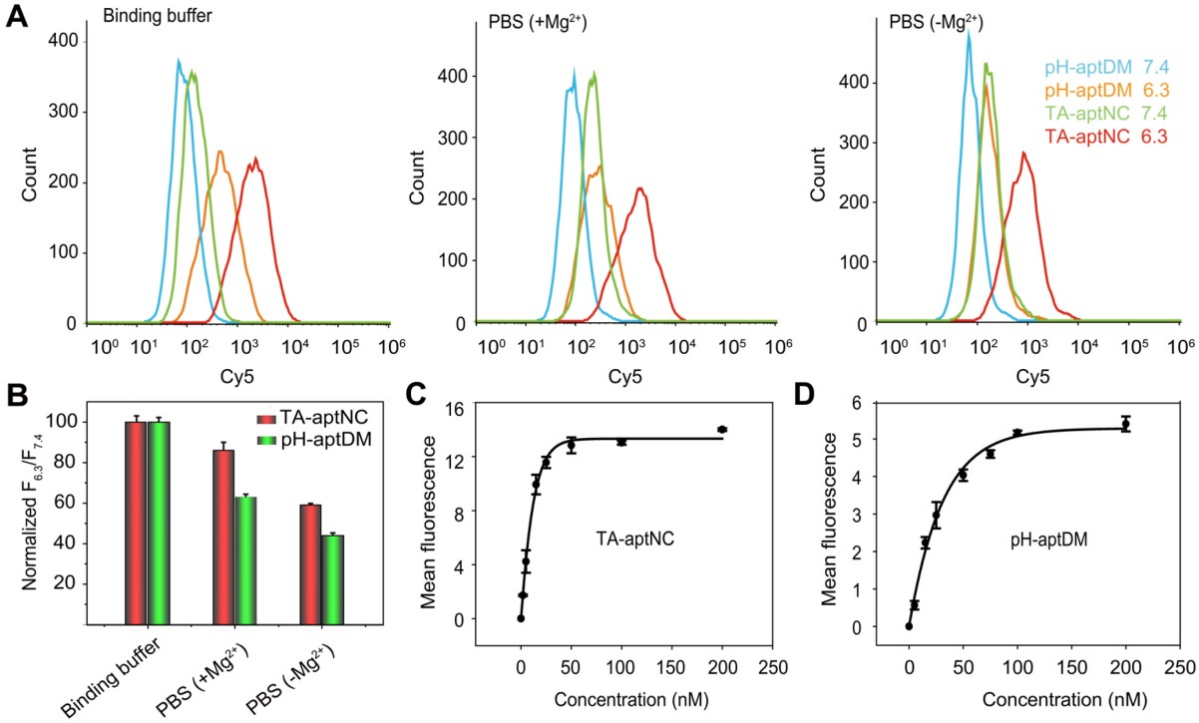
** Flow cytometry analysis of the binding performance of multivalent TA-aptNC, in comparison with monovalent pH-aptDM. (A)** The binding stability of TA-aptNC in binding buffer, PBS (+ Mg^2+^ ) and PBS (- Mg^2+^ ). **(B)** Histogram of the corresponding normalized signal ratios of SMMC-7721 cells at pH 6.3 to at pH 7.4 for TA-aptNC in (A). Dissociation constant of TA-aptNC **(C)** and pH-aptDM **(D)** against target SMMC-7721 cells. Data are represented as means ± SD (n = 3).

Compared to the pH-aptDM, the resultant TA-aptNC was desired to keep relatively stable affinity to target cells. Subsequently, the binding capacity of TA-aptNC was explored in some typical buffers, including binding buffer (provided yeast-tRNA, Mg^2+^, glucose and BSA into PBS), PBS with Mg^2+^ provided (+ Mg^2+^) and PBS without Mg^2+^ provided (- Mg^2+^). The flow cytometry assays indicated that a high binding affinity was in binding buffer, by comparing with that in other buffers, which is due to that the majority of aptamers were selected via cell-SELEX in binding buffer (**Figure 3A-B**). When removing bivalent cationic, monovalent pH-aptDM displayed an ~60% drop of signal-to-background ratio (SBR). Surprisingly, the binding ability of TA-aptNC could still maintained ~60% of that in binding buffer. We can conclude that the binding stability of TA-aptNC is superior to that of pH-aptDM, which might be due to the multivalent effect and the increase in mass accompanied by the reduction of conformational energy penalty. The stable binding capacity would greatly promote the application of the TA-aptNC in real biological systems.

Binding affinity of aptamers is an important parameter for evaluating the binding performance of diagnostic and/or therapeutic probes to target cells. In this study, the multivalent binding ability of TA-aptNC was dependent on the multiple aptamer nanoclaw embedded in the three outstretched arm of scaffold. The binding affinity of TA-aptNC against target cancer cells was evaluated by flow cytometric analysis, and quantified by the equilibrium dissociation constant (Kd, which is inversely related to the binding affinity). The TA-aptNC activated at acidic pH exhibited a better binding affinity (Kd = 9.00 ± 0.60 nM, **Figure 3C**), which was 2 times higher than that of pH-aptDM (Kd = 18.00 ± 0.10 nM, in **Figure 3D**).

### Sensitivity and selectivity of the TA-aptNC-based *in situ* assembly system

In view of the *in situ* assembled TA-aptNC with superior binding affinity, the sensitivity of the proposed strategy was evaluated by quantitative analysis of SMMC-7721 cells. The samples dispersed with different number of cells ranging from 16 to 160000 were prepared by gradient dilution in 200 μL of binding buffer. The flow cytometry assays indicated that the TA-aptNC-labelled SMMC-7721 cells locating in the upper right region decreased gradually with the decrease of cell number **(Figure S8A)**. An excellent linear relationship was obtained in the range of 16 to 160000 cells. The calibration equation was y = 0.865x - 15.284 with a correlation coefficient R^2^ = 0.9989 **(Figure S8B).** The lowest number of SMMC-7721 cell detected by this strategy was 16 in 200 μL of sample, showing the excellent sensitivity of this strategy.

The accurate recognition of tumor has presented a great challenge not only in sensitivity but more importantly in selectivity. To indicated that TA-aptNC-based *in situ* assembly system could specifically recognize target SMMC-7721 cells, normal L02 cells and three kinds of cancer cells (HepG2, HeLa and MCF-7 cells) were chosen to investigate the specificity of this strategy. As shown in **Figure S9,** the mixture of three DNA monomers (TA-aptNC group) presented an obvious fluorescence labelling to SMMC-7721 cells at pH 6.3. By contrast, negligible fluorescence signals in response to pH changes were observed for the normal cell and other three tumor cells. To further confirm the above results from flow cytometry, we examined the TA-aptNC for distinguishing of target cell using confocal microscopy **(Figure S10)**. Bright red halos around cells were only observed for target SMMC-7721 cells after incubating with the mixture of three pH-aptDMs at pH 6.3. Whereas there was almost no obvious Cy5 fluorescence in negative control cells, which was in accord with the results of flow cytometry analysis. The great sensitivity and specificity promised the potential application of TA-aptNC-based *in situ* assembly system in accuracy cancer diagnosis and therapy.

### Cytotoxicity and cell uptake behavior of the TA-aptNC-based *in situ* assembly system *in vitro*

Considering the *in situ* assembled TA-aptNC with significant increased binding to target cells, we envisioned that it could be evaluated as targeted drug delivery carrier for enhancing cancer therapy. In this study, Dox as a drug model was preferentially inserted into GC base pairs of three pH-aptDMs with quenched fluorescence. Accordingly, the drug payload performance of the DM_1I_, DM_2I_ and DM_3I_ were evaluated by the fluorescence spectrum, respectively. As shown in **Figure S11**, the Dox signals were gradually decreased as pH-aptDM concentration increased and reached a saturation at a molar radio of 1:5 (pH-aptDM/Dox). Then, we ascertain the stability and drug release of the Dox-loaded TA-aptNC (TA-aptNC-Dox) at physiological temperature** (Figure S12A)**. The TA-aptNC-Dox and pH-aptDM-Dox both displayed desirable stability in PBS, and a slight drug leakage in 10% fetal bovine serum. However, when both of Dox-loaded probes diffusing in high dose of nuclease, Dox was successfully released with strong fluorescence signals owing to hydrolysis of DNA nanocarriers **(Figure S12B).** However, TA-aptNC-Dox showed more slower fluorescence restoration than pH-aptDM-Dox, suggesting the excellent stability of TA-aptNC in intracellular drug delivery.

**Figure 4:**
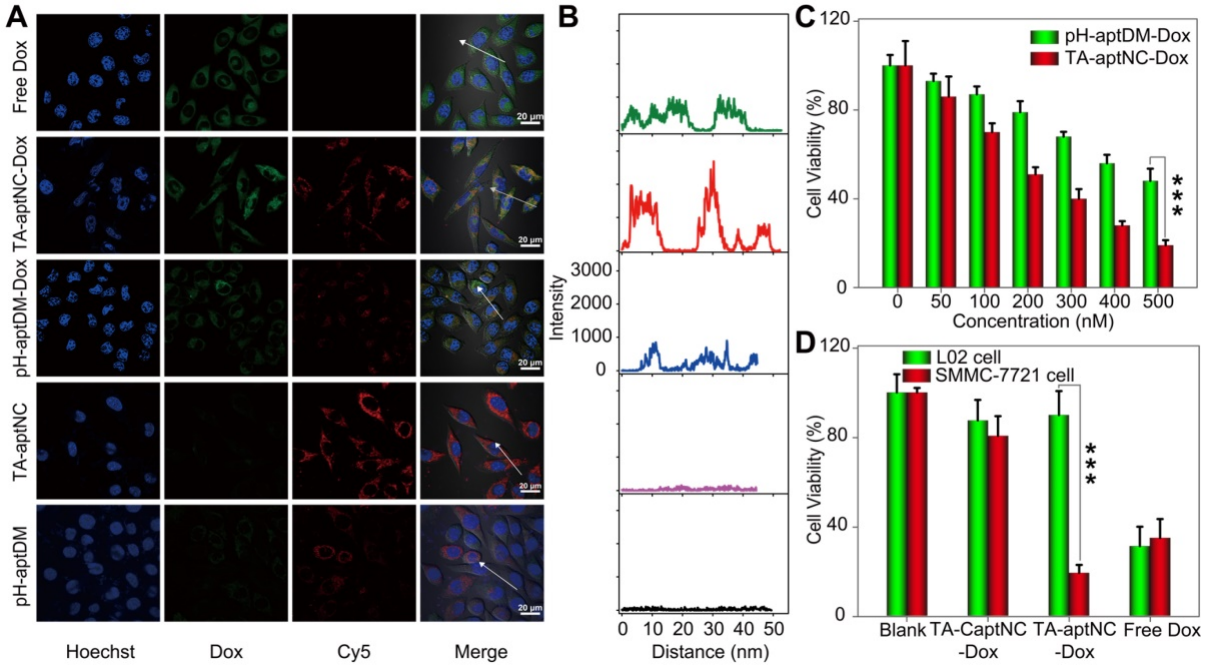
**(A)** Confocal laser scanning microscopy images of target SMMC-7721 cells after treatment with different compositions, including free Dox, TA-aptNC-Dox (the mixture of DM_1I_-Dox, DM_2I_-Dox and DM_3I_-Dox), pH-aptDM-Dox, TA-aptNC (the mixture of DM_1I_, DM_2I_ and DM_3I_) and pH-aptDM in binding buffer at pH 6.3 (scale bar: 20 μm). **(B)** The corresponding diagrams show the Dox fluorescence intensity on the arrows drawn in the images (A). **(C)** Cytotoxicity investigation of target tumor cells after incubation with the mixture at pH 7.4 (pH-aptDM-Dox) or pH 6.3 (TA-aptNC-Dox) for 48 h at different doses. **(D)** The selective cytotoxicity of target tumor cells and normal L02 cells after treated with different formulations. Data are represented as means ± SD (n = 3). The *p* values were calculated by the Student's *t* test (***p < 0.001).

Subsequently, we investigated the time dependent internalization of Cy5-labelled TA-aptNC (Cy5-TA-aptNC) into target cells (**Figure S13**). The results showed an enhanced intracellular fluorescence signal with the increase of culture time from 0.5 to 4 h and a plateau at about 3 h. Further, we evaluated the drug delivery and release capacity of the TA-aptNC in target cells. As shown in **Figure 4A-B**, the brighter fluorescence signal of Cy5 and Dox were observed by the TA-aptNC-Dox treated group (the mixture of three Cy5-pH-aptDMs-Dox incubated with SMMC-7721 cells at pH 6.3), in comparison with the pH-aptDM-Dox treated group under the same condition. Presumably, at pH 6.3, the mixture of the three pH-aptDM-Dox was assembled into TA-aptNC-Dox, which could aggregate on the target cell surface, thus facilitating cellular internalization with the aid of surface-anchored multiple aptamer nanoclaw. In the effect of pH, ionic and ubiquitous nucleases present in cytoplasm, Dox was released from the resulting TA-aptNC-Dox with significant fluorescence recovery. The results demonstrated that acidic pH successfully activated the assembly of TA-aptNC-Dox with an improved drug delivery efficiency at tumor site. Meanwhile, the selective drug uptake behavior was studied with target SMMC-7721 cells and notarget L02 cells (**Figure S14**). An obvious Dox fluorescence signal was observed in SMMC-7721 cells treated with the mixture at pH 6.3 than that of at pH 7.4. It is due to that the acquired multivalent effect could facilitate internalization of TA-aptNCs-Dox into target tumor cells through aptamer-receptor interaction with improved drug delivery efficience. Moreover, when L02 cells were treated with the mixture at pH 6.3, the formation of i-motif led to the release of partial Dox from the pH-aptDMs-Dox. Therefore, a relatively weak Dox fluorescence signal was observed only at pH 6.3 but not at 7.4. It indicates the highly selective drug delivery mediated by TA-aptNC-based *in situ* assembly system.

Having established that TA-aptNC-Dox could be selectively internalized into cells and realize intracellular drug release, we next assessed the selective cytotoxicity of the TA-aptNC-Dox using MTS assays. As presented in **Figure 4C**, both the mixture of three Dox-loaded monomer (TA-aptNC-Dox group) and the pH-aptDM-Dox could induce cell apoptosis in a dose-dependent manner. The resulting multivalent effect could accelerate the internalization of the *in situ* assembled TA-aptNC-Dox into target SMMC-7721 cells, thereby enabling the enhanced cytotoxicity. Furthermore, benefiting from the enhanced affinity and excellent specificity, the TA-aptNC-Dox showed robust cytotoxicity on target cells rather than nontarget cells at pH 6.3 (**Figure 4D**). In contrast, free Dox displayed indiscriminate cytotoxicity for both SMMC-7721 cells and L02 cells, indicating the excellent selective cytotoxicity of the TA-aptNC. As anticipated, the pure TA-aptNC and pH-aptDM displayed a negligible cytotoxicity for target cells either, suggesting good biocompatibility of the TA-aptNC-based *in situ* assembly strategy (**Figure S15**). In general, the selective and potent* in vitro* therapeutic efficacy of TA-aptNC might pave the way for precise therapy *in vivo*.

### *In vivo* imaging of the TA-aptNC-based* in situ* assembly system

**Figure 5:**
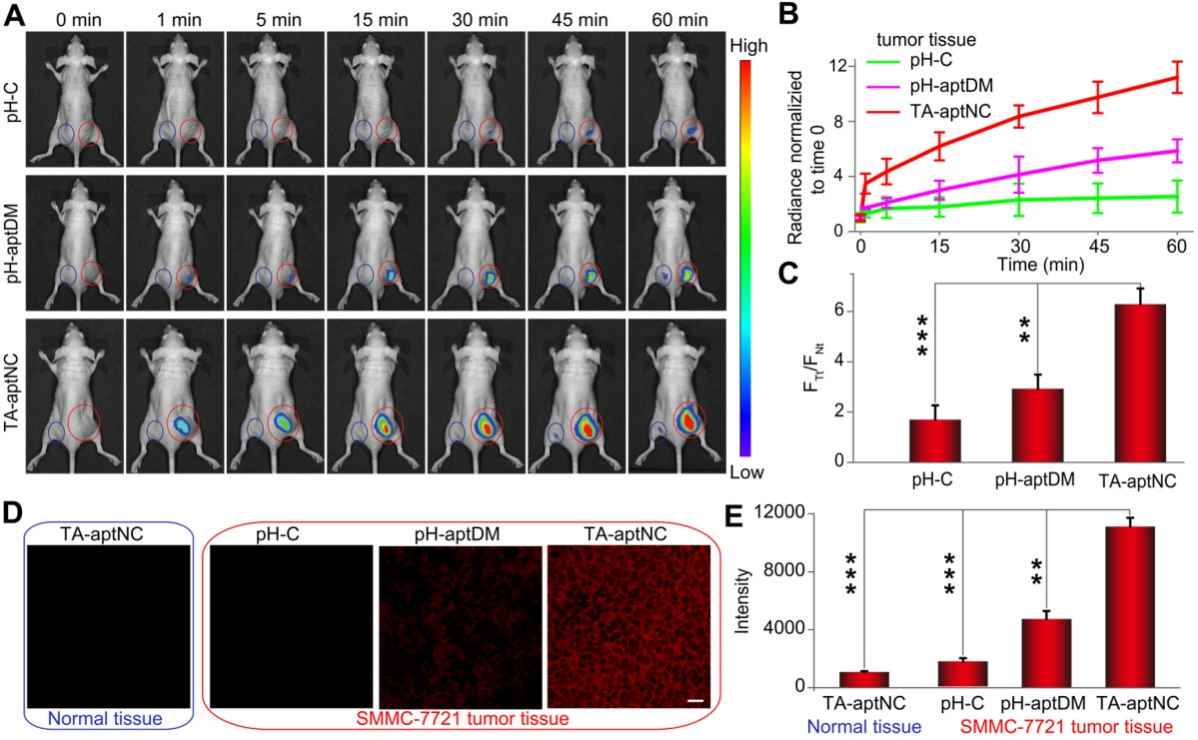
**Investigation of acidic tumor microenvironment specificlly activated *in vivo* tumor imaging by the *in situ* assembled multivalent nanodevice. (A)** Time-dependent *in vivo* fluorescence imaging monitoring of the SMMC-7721 tumor-bearing nude mice after intratumoral injection of pH-C, pH-aptDM and TA-aptNC (the mixture of three pH-aptDM monomers), respectively. The corresponding probes treated normal tissues were imaged as controls (Normal tissue: blue circle; SMMC-7721 tumor: red circle.). **(B)** Quantification of the fluorescence intensity at the tumor sites and normal tissues in (A). **(C)** Analysis of signal intensity ratios of tumor tissues (F_Tt_) to normal tissues (F_Nt_) for different probes in (A) at 1 h. **(D)** Confocal laser scanning microscopy images of the tumor slices and normal tissue slices after incubation with different probes (scale bar: 200 μm). **(E)** The corresponding histogram of the four compositions show the mean fluorescence intensity in (D). Data are represented as means ± SD (n = 3). The *p* values were calculated by the Student's *t* test (***p < 0.001, **p < 0.01).

After verifying the TME-activated contrast-enhanced imaging effect and selective cytotoxicity to target SMMC-7721 cells *in vitro*, we evaluated the utility of the proposed strategy *in vivo* wherein acidic tumor microenvironment could guide *in situ* assembly of three pH-aptDMs into TA-aptNC for contrast-enhanced tumor imaging. pH-C, pH-aptDM and the mixture of three monomers (TA-aptNC group), under the same condition, was injected into the tumor site (red circle) of SMMC- 7721 tumor-bearing nude mice, respectively. Simultaneously, the corresponding normal tissues (blue circle) were treated by a same method as negative controls for time-dependent *in vivo* fluorescence images (**Figure 5A**). Both the TA-aptNC group and pH-aptDM group established an increased intratumoral fluorescence with the time extended as a result of the continuous activation of fluorescence signals by acidic tumor microenvironment. Significantly, unlike pH-aptDM, TA-aptNC group realized a fast, accuracy and strong fluorescence recovery at tumor site as early as 1 min after intertumoral injection. However, the control groups (pH-C-treated tumor and all the corresponding normal tissues treated with different probes) displayed an ignorable fluorescence enhancement. Quantification analysis indicated that TA-aptNC group displayed outstanding fluorescence contrast (F_Tt_/F_Nt_, the fluorescence signals ratio of the same probe treated with tumor tissues to corresponding normal tissues), which was 3.8-fold higher than that of pH-C group at 60 min (**Figure 5B-C**). While the fluorescence contrast of monovalent pH-aptDM group was merely 1.8 times of pH-C. We thus deduced that three monomers in the mixture could competitively bind with each other to promote the release of BHQ2 labelled split-I from Cy5 labelled S at complex acidic tumor microenvironment, thus generating significant activatable intratumoral signals. Consistent with the results *in vitro*, the results in animal model highlighted that this strategy could be high-efficiently activated in TME but remains silence in normal tissues, which is of great importance for sensitive and accurate tumor diagnosis.

The contrast-enhanced tumor imaging was further investigated by imaging frozen tumor sections and normal tissue sections. As presented in **Figure 5D**, a negligible fluorescence signal was observed for the pH-C treated tumor sections and all of normal tissue sections. Note that TA-aptNC group showed stronger capability to image heterogeneous tumor microenvironments than pH-aptDM-treated group. Taken together, the *in situ* activated multivalent nanodevice (TA-aptNC) showed accurate and contrast-enhanced imaging capacity in extremely complex tumor environment without any auxiliary additives.

### *In vivo* antitumor effect of the pH-triggered TA-aptNC-based *in situ* assembly system

**Figure 6:**
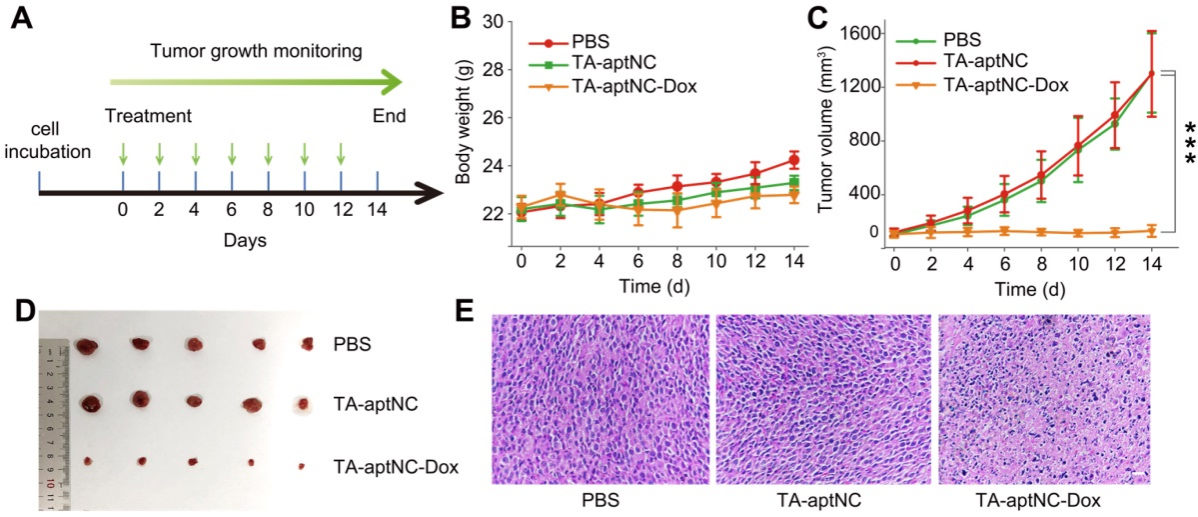
***In vivo* tumor growth inhibition study of the TA-aptNC-based *in situ* assembly system. (A)** The SMMC-7721 tumor-bearing BALB/c nude mice model was established at day 0. The mice were randomly divided into three groups and treated with various formulations (PBS, TA-aptNC, TA-aptNC-Dox (the mixture of three pH-aptDMs)) at days 0, 2, 4, 6, 8, 10 and 12. The body weight curve **(B)** and the tumor volume curve **(C)** of nude mice treated with various formulations, respectively. **(D)** Photographs of dissected tumors in various treatment groups. **(E)** H&E staining images of tumor tissues in all treatment groups (scale bars: 200 μm). The *p* values were calculated by the Student's *t* test (***p < 0.001).

We subsequently evaluated its antitumor performance *in vivo* using nude mice bearing SMMC-7721 xenograft tumor. All tumor-bearing BALB/c mice were randomly divided into three groups (n = 5), and treated with PBS, the mixture of three monomers (TA-aptNC group) and the mixture of Dox-loaded three monomers (TA-aptNC-Dox group) every two days by intratumoral injection, respectively. The time schedule of the treatment process was shown in **Figure 6A**. During the animal experiment, the body weight of each nude mice was stable without a significant fluctuation **(Figure 6B)**, demonstrating the limited systemic toxicity and the excellent biocompatibility of *in situ* assembled TA-aptNC. The real-time variations of tumor volume were also recorded **(Figure 6C)**. The continuous increase of tumor volume was observed for the mixture of three monomers injected mice (TA-aptNC group) in similar with the PBS-treated control group, indicating almost no inhibition of tumor growth, which excluded the systemic toxicity of our proposed strategy. Noteworthy, the TA-aptNC-Dox group displayed a high antitumor efficacy with 90% inhibition. The growth inhibition of tumor was clearly validated by the photographs of dissected tumors for different treatment groups **(Figure 6D)**. In addition, the hematoxylin and eosin (H&E) staining also confirmed that the TA-aptNC group caused a significant disruption of nucleus structures and tumor tissue morphology **(Figure 6E)**, in accordance with the outcome of abovementioned tumor inhibition. Collectively, these results showed that *in situ* assembly of precisely controlled TA-aptNC afforded superior antitumor efficacy and a negligible systemic toxicity.

### Biocompatibility evaluation

Biocompatibility of drug deliverer is regarded as a requisite in biomedical applications. The hemolysis experiment was performed to investigate the *in vivo* biocompatibility of this system. First, the important hematological biomarkers of rat red blood cells (RBC) were treated with ultrapure H_2_O and PBS as the positive control and negative control, respectively. As shown in **Figure S16**, there was no significant hemolysis after incubating RBCs with different concentrations of the mixture of three pH-aptDMs range from 1 to 150 μM for 12 h at 37 °C, even at high concentrations of the mixture of three pH-aptDMs, indicating the excellent blood compatibility of the TA-aptNC system. The hematological analyses further revealed the exceptional biocompatibility and robust *in vivo* applicability of this system.

## Conclusion

In summary, endogenous stimuli-powered *in situ* assembly of precisely controlled activatable multivalent nanodevice (TA-aptNC) in an extremely complex tumor microenvironment was engineered for facilitating their imaging function and antitumor efficiency. In comparison with the monovalent DNA monomer or static assembled multivalent DNA nanotheranostics system, the proposed strategy possessed some superiorities: (1) The activated TA-aptNC, possessed the unique superiorities of the precise controlled spatial orientation, defined ligand density and structure size, which allowed 2-fold enhanced binding ability and improved endocytosis efficiency, thus enabling the TME-activated contrast-enhanced *in vivo* tumor imaging and significant growth inhibition of tumor. TA-aptNC-based *in situ* assembly system could minimize the background signal in nonspecific site and exclude a risk of irreversible damage to normal tissues. (2) Irrespective of the recruitment of any exogenous biological or nonbiological auxiliary additives, the assembly of TA-aptNC imitated naturally occurring process at a certain location, which might offer unprecedented opportunity to understand the mechanism of disease occurrence and guide the therapy precisely. The strategy could be universally applicable to different tumor by altering targeted identification primitives, which broadened its application scope in various diseases. The proposed strategy will open new avenue for modulating biological behavior in specific site, laying a foundation for the future accurate diagnosis and therapeutics.

## Supplementary Material

Supplementary materials and methods, figures and table.

## Abbreviations

TA-aptNC: three-arm aptamer nanoclaw
pH-aptDMs: pH-responsive aptamer-decorated DNA monomers
DM: DNA module
TA-aptNC-Dox: Dox-loaded TA-aptNC
pH-aptDM-Dox: Dox-loaded pH-aptDM
Cy5-TA-aptNC: Cy5 labeled TA-aptNC
Cy5- pH-aptDM: Cy5 labeled pH-aptDM
PAGE: polyacrylamide gel electrophoresis
AFM: atomic force microscopic atomic force microscopic
RCA: rolling circle amplification
TME: tumor microenvironment
MTT: methyl thiazolyl tetrazolium
SNR: signal-to background ratio
H&E: hematoxylin and eosin
