# Supplementary material for: Acidic microenvironment triggered in situ assembly of activatable three-arm aptamer nanoclaw for contrast-enhanced imaging and tumor growth inhibition in vivo
Source: Theranostics. 2022 Apr 24;12(7):3474–87. doi: 10.7150/thno.72028 (PMC9065194; doi:10.7150/thno.72028)
Supplement: Supplementary file 1 — Supplementary materials and methods, figures and table. [file thnov12p3474s1.pdf]

Supporting information

Acidic microenvironment triggered *in situ* assembly of activatable three-arm aptamer nanoclaw for contrast-enhanced imaging and tumor growth inhibition *in Vivo*

Jin Huang, Yuchen Wu, Hui He, Wenjie Ma, Jianbo Liu, Hong Cheng, Huanhuan Sun, Xiaoxiao He*, Kemin Wang*

State Key Laboratory of Chemo/Biosensing and Chemometrics, College of Biology, College of Chemistry and Chemical Engineering, Hunan University, Key Laboratory for Bio-Nanotechnology and Molecule Engineering of Hunan Province, Changsha 410082, China. Email: xiaoxiaohe@hnu.edu.cn, kmwang@hnu.edu.cn.

Table of Contents

Experimental procedures 2

Reagents and instruments 2

Atomic force microscopy imaging 2

CD measurement........ 3

Fluorescence experiment 3

Binding affinity investigation 3

Investigation of the specificity........ 4

Cell culture........ 5

Cellular internalization investigation........ 5

Statistical analysis........ 6

Table 1 6

Figures S1-S16........ 7

References........ 20

Experimental Procedures

Reagents and Instruments.

The oligonucleotides used in this study were synthesized and HPLC purified by Sangon Biotechnology Co., Ltd. (Shanghai, China). Sequences of oligonucleotides were listed in Table S1 (Supporting Information). Deoxyribonuclease I (DNase I) was purchased from New England Biolabs LTD (Beijing, China). Bull serum albumin (BSA) and yeast tRNA were purchased from Sigma Aldrich. Dulbecco's phosphate buffered saline (D-PBS), SYBR Gold, Dulbecco's Modified Eagle Medium (DMEM) were obtained from Thermo Fisher Scientific (MA, U.S.A). 6 × Loading buffer was obtained from TaKaRa Bio Inc. (Dalian, China). Doxorubicin (Dox) was obtained from Hualan Chemistry Technology Co. Ltd. Optimal Cutting Temperature Compound (OCT) was purchased from SAKURA (Torrance, U.S.A). Hoechst 33342 was purchased from Sangon Biotechnology Co., Ltd. (Shanghai, China). The MTS Cell Proliferation Colorimetric Assay Kit was purchased from Promega (WI). Phosphate buffered saline (10 mM PBS) with different pH value was prepared by mixing 10 mM $Na_2HPO_4 \cdot 12H_2O$, 10 mM $NaH2PO_4 \cdot 2H_2O$, 130 mM NaCl, 4.6 mM KCl and 5 mM $MgCl_2$. Binding buffer was prepared by adding $MgCl_2$ (5 mM), BSA (1 mg/mL), glucose (4.5 mg/mL) and yeast tRNA (0.1 mg/mL) into D-PBS. MTT assays were conducted on a M1000 microplate reader (TECAN Inc., America). The pH of all solutions was calibrated with a San-Xin MP511 miniature pH meter (Shanghai, China). Centrifugation was performed on Beckman Coulter Allegra 25R centrifuge (America). Fluorescence spectra were recorded using a Hitachi F-7000 fluorescence spectrometer (Japan). Ultrapure water (Milli-Q 18.2 MΩ, Millipore System Inc.) was used for all the experiments. Gel imaging was conducted on Azure C600 (America) multifunctional molecular imaging analysis system. All other reagents were of the highest grade available and obtained from Sinopharm Chemical Reagent Co. Ltd. (Shanghai, China).

Atomic Force Microscopy (AFM).

pH-triggered formation of the TA-aptNC was characterized by AFM (SEIKO, Japan). Samples for AFM imaging were prepared by diluting the mixture of three pH-aptDMs

to ~25 nM in PBS (pH 7.4 or 6.3). After dilution, 20 μL of sample solution was deposited onto a freshly cleaved mica surface (Ted Pella, Inc.) for 10 min and then washed three times with deionized water. Last, the samples were scanned using a tapping mode.

CD Measurement.

300 μL of probes (I_1, ZYsls-C, pH-aptDM and the mixture of DM_{1I}, DM_{2I} and DM_{3I}) were incubated in PBS with different pH values for 1 h at 37 °C. Then, circular dichroism (CD) spectra of each sample were acquired at room temperature in the range of 220-320 nm by Bio-Logic MOS-500 CD spectrophotometer (Claix, France). In each case, three scans were performed and averaged after subtracting the background of the corresponding PBS solution. The final concentration of all sample was 2.5 μM.

Fluorescence Experiment.

To investigate the fluorescence stability of Cy5 at different pH, fluorescence spectra of Cy5 labeled S_1, Cy5 labeled S_2 and Cy5 labeled S_3 were monitored by an F-7000 fluorescence spectrophotometer. In order to characterize the pH-responsive performance, we first optimize the assembly of three pH-aptDMs by altering the ratio of BHQ2-I to Cy5-S. Then, the formation of pH-aptDMs was determined on an F-7000 fluorescence spectrophotometer (Hitachi, Japan) by measuring the fluorescence signal of Cy5-labeled S. The DM_{1I}, DM_{2I}, DM_{3I} and the mixture of three pH-aptDMs in response to the pH changes were also measured at 37 °C using an F-7000 fluorescence spectrophotometer. The final concentration of all samples were100 nM. (Ex: 620 nm; Em: 640-740 nm).

Binding affinity investigation.

The binding affinity of TA-aptNC (the mixture of DM_{1I}, DM_{2I} and DM_{3I}) or pH-aptDM was determined by incubating SMMC-7721 cells with varying concentrations of the corresponding probes in 200 μL binding buffer at 37 °C for 60 min. TA-CaptNC treated group was used as negative controls to determine nonspecific binding. After incubation, the DNA probes treated target cells were then washed 2 times and re-dispersed in PBS.

Last, the cells were subjected to flowcytometric analysis. Data were analyzed with the FlowJo software. The mean fluorescence intensity of SMMC-7721 cells labeled by different probes was used to calculate for specific binding by subtracting the mean fluorescence intensity of nonspecific binding from the control probe. The equilibrium dissociation constants (Kd) of the aptamer-integrated probes-cell interaction was obtained by fitting the dependence of fluorescence intensity of specific binding on the concentration of the aptamer-integrated probes to the equation $Y = Bmax X / (Kd + X)$, where Y is the fluorescence intensity and X is the concentration of aptamers, using SigmaPlot.

Investigation of the specificity.

The mixture of three pH-aptDMs incubated with different cell lines (target SMMC-7721 cells, HeLa cells, HepG2 cells, MCF-7 cells and L02 cells) in different pH at 37 °C for 1 h, respectively. The cells were analyzed with a Gallios cytometer. For the sensitivity analysis of this strategy, the same concentration mixture of three pH-aptDMs were respectively incubated with various cell numbers ranging from 0 to 160000 in 200 μL of binding buffer. After incubation, all samples were monitored on a Gallios cytometer by collecting the fluorescence signal in FL6 for Cy5 (fluorescence channel Ex = 633 nm, Em = 660 nm band-pass). All experiments were independently repeated three times.

Dox Loading and Release Investigation. A fixed concentration of Dox (1 μM) was mixed with various concentrations of prepared DM_{1I}, DM_{2I} and DM_{3I} in PBS at room temperature for 1 h. The Dox loading capacity of each pH-aptDM monomer were monitored by the fluorescence signal of Dox using an F-7000 fluorescence spectrophotometer. To evaluate the stability and Dox unloading efficiency of the Dox loaded pH-aptDM (pH-aptDM-Dox) and Dox loaded TA-aptNC (TA-aptNC-Dox), pH-aptDM or TA-aptNC and Dox (mole rations of 1:5) were firstly mixed to prepare pH-aptDM-Dox and TA-aptNC-Dox as described above. subsequently, these complexes of DNA-Dox were dispersed into PBS buffer, PBS buffer containing 10% FBS and PBS buffer containing DNase I (1.5 U/mL), respectively. The real-time fluorescence

intensities were monitored by an F-7000 fluorescence spectrophotometer. (Ex = 488 nm, Em = 520-740 nm).

Cell culture.

SMMC-7721 cells (human hepatoma cell line), HeLa cells (human cervical cancer cell line) and L02 cells (normal liver cell line) were cultured in RPMI 1640 medium. HepG2 cells (Human hepatocellular carcinoma cell line) and MCF-7 cells (human breast cancer cell line) were cultured in Dulbecco's modified Eagle medium (DMEM). Both cultures were supplemented with 12% fetal bovine serum (FBS), 100 U/mL penicillin and 100 μg/mL streptomycin, and maintained at 37 °C in a 5% CO_2 atmosphere in humidified HF90 CO_2 incubator (Shanghai Lishen Scientific Equipment Co., Ltd.). All of cells used in this study were obtained from the Cell Bank of the Committee on Type Culture Collection of the Chinese Academy of Sciences (Shanghai, China). The cell density was determined by hemocytometer before any cell experiments.

Cellular internalization investigation.

To evaluate the time-dependent internalization of Cy5 labeled TA-aptNC (Cy5-TA-aptNC), SMMC-7721 cells were dispersed on 35-mm confocal incubation dishes for 24 h. The mixtures of Cy5 labeled DM_{1I}, DM_{2I} and DM_{3I} were respectively incubated with SMMC-7721 cells at 37°C in CO_2 incubator for 0.5, 1, 2, 3 and 4 h. Subsequently, the fluorescence imaging was performed on Nikon A1 confocal laser scanning microscopy (CLSM).

Supplemental Materials

Table 1. All oligonucleotide sequences in this work (from 5' to 3')

Name	Sequence (5'-3')
**ZYsls-C**	ATCTCGGAGCCACGCTTTTTTTTTTACGCGCGCGCGCATAG CGCGCTGAGCTGAAGATCGTACCGTGAGCGCGT
**Control**	ATCTCGGAGCCACGCTTTTTTTTTTN49
**S_1**	GCGTGGCTCCGAGATATACTCATCCTGTTGAC GTAT
**S_2**	GCGTGGCTCCGAGATTAGCACATGCTGGATGAGTAT
**S_3**	GCGTGGCTCCGAGATATACGTCAACTGCATGTGCTA
**I_1**	CCCCCCTCCCCCCTACGTCAACAGGCTCCCCCCTCCCCCC
**I_2**	CCCCCCTCCCCCCTACTCATCCAGCCTCCCCCCTCCCCCC
**I_3**	CCCCCCTCCCCCCTAGCACATGCAGCTCCCCCCTCCCCCC
**IC_1**	TTTTTTTTTTTTTTACGTCAACAGGCTTTTTTTTTTTTTT
**IC_2**	TTTTTTTTTTTTTTACTCATCCAGCCTTTTTTTTTTTTTT
**IC_3**	TTTTTTTTTTTTTTAGCACATGCAGCTTTTTTTTTTTTTT
**Cy5-S_1**	GCGTGGCTCCGAGATATACTCATCCTGTTGAC GTAT-Cy5
**Cy5-S_2**	GCGTGGCTCCGAGATTAGCACATGCTGGATGAGTAT-Cy5
**Cy5-S_3**	GCGTGGCTCCGAGATATACGTCAACTGCATGTGCTA-Cy5
**BHQ2-I_1**	CCCCCCTCCCCCC-(BHQ2)-TACGTCAACAGGCTCCCCCCTC CCCCC
**BHQ2-I_2**	CCCCCCTCCCCCCT-(BHQ2)-ACTCATCCAGCCTCCCCCCTC CCCCC
**BHQ2-I_3**	CCCCCCTCCCCCCT-(BHQ2)-AGCACATGCAGCTCCCCCCTC CCCCC
**BHQ2-IC_1**	TTTTTTTTTTTTTT-(BHQ2)-ACGTCAACAGGCTTTTTTTTTT TTT
**BHQ2-IC_2**	TTTTTTTTTTTTTT-(BHQ2)-ACTCATCCAGCCTTTTTTTTTT TTT
**BHQ2-IC_3**	TTTTTTTTTTTTTT-(BHQ2)-AGCACATGCAGCTTTTTTTTTT TTT

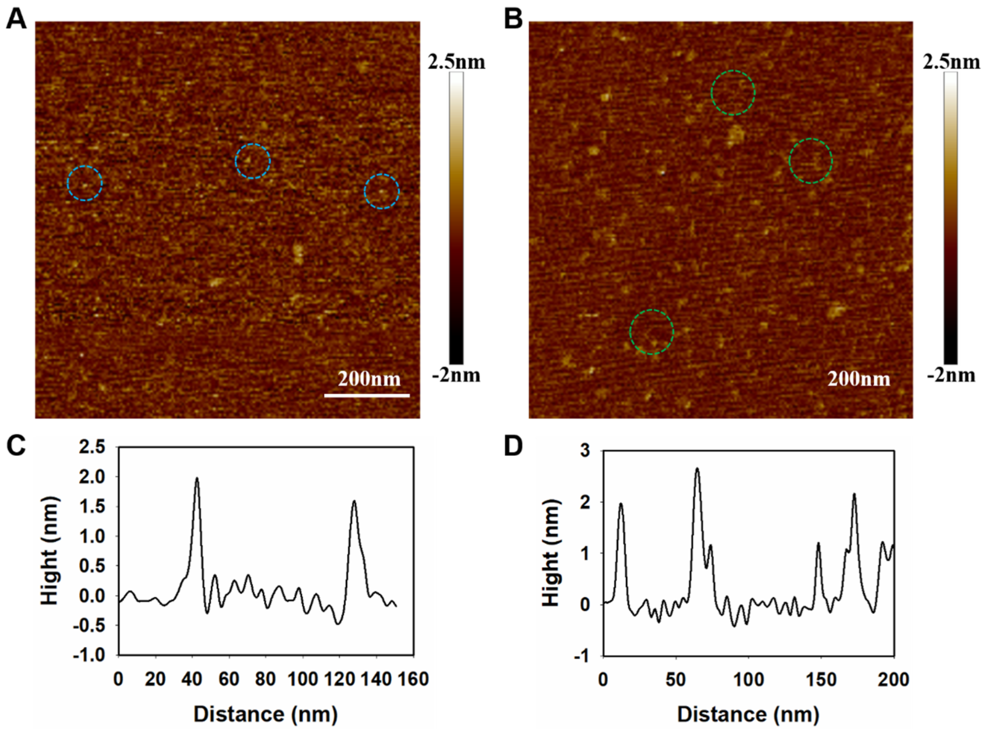

Figure S1. AFM images of the mixture of three pH-aptDMs at pH 7.4 (A) and pH 6.3 (B). The sizes of pH-aptDM and TA-aptNC are shown in (C) and (D), respectively.

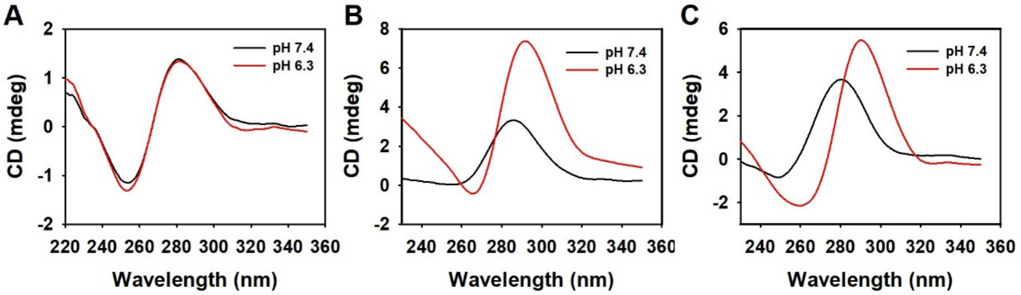

Figure S2. The CD spectra of various probes recorded in 10 mM PBS buffer with different pH at 37 °C. (A) ZYsls-C only; (B) split-I-motif only; (C) monodispersed pH-aptDM. Probe concentration: 2.5 μM. The CD spectral profile characterized with a positive band at ~270 nm and a negative band at ~253 nm was respectively redshifted to ~290 and ~265 nm, indicating the formation of i-motif structures.

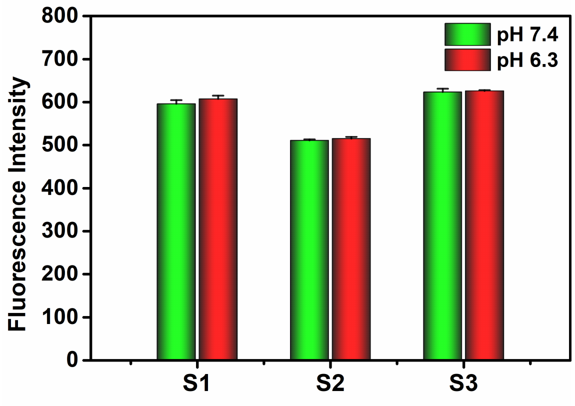

Figure S3. Fluorescence stability investigation of Cy5 labeled S_1, Cy5 labeled S_2 and Cy5 labeled S_3 (500 nM) in 10 mM PBS buffer with different pH values at 37 ^oC for 60 min.

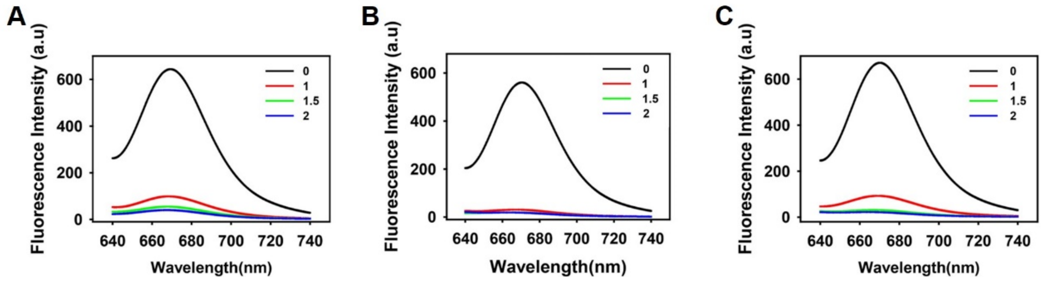

Figure S4. The fluorescence spectra characterization of the assembly of three DNA modules (ZYsls-C + Cy5-S + BHQ2-I): (A) DM_{1I}; (B) DM_{2I}; and (C) DM_{3I} by altering the ratio of BHQ2-I to Cy5-S at 37 ^{o}C for 60 min. Probe concentration: 100 nM.

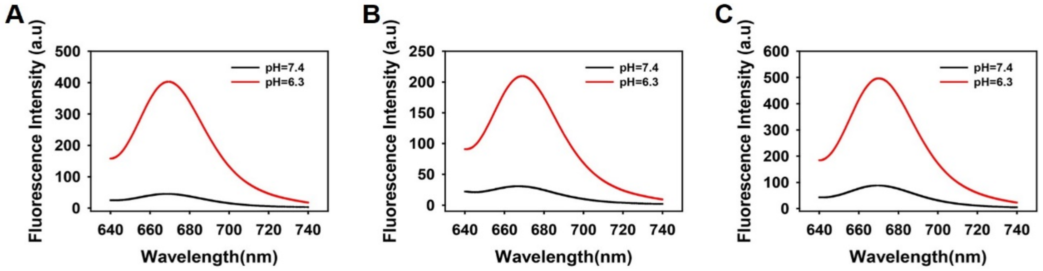

Figure S5. The fluorescence spectra characterization of the pH-responsiveness of individual DNA module (A) DM_{1I}; (B) DM_{2I}; and (C) DM_{3I} in 10 mM PBS buffer with different pH values at 37 ^{o}C. Probe concentration: 100 nM.

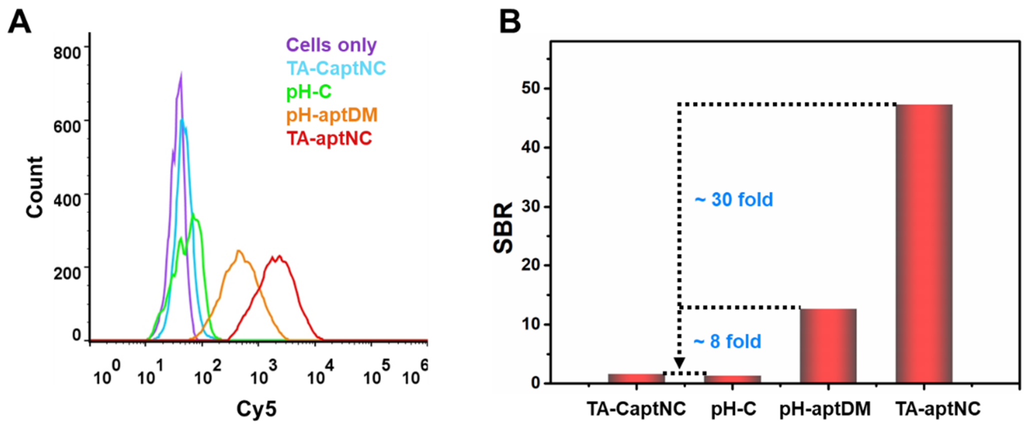

Figure S6. (A) Flow cytometric analysis of SMMC-7721 cells treated with different combinations in binding buffer at pH 6.3, respectively. (B) The corresponding signal-to-background ratio (SBR) of TA-CaptNC group (the mixture of CDM_{1I}, CDM_{2I} and CDM_{3I}), pH-C group (the mixture of DM_{1IC}, DM_{2IC} and DM_{3IC}), pH-aptDM group (the corresponding monovalent probe) and TA-aptNC group (the mixture of DM_{1I}, DM_{2I} and DM_{3I}) in (A) for detecting SMMC-7721 cells at 37 ^{o}C for 60 min. (Probe concentration: 100 nM.)

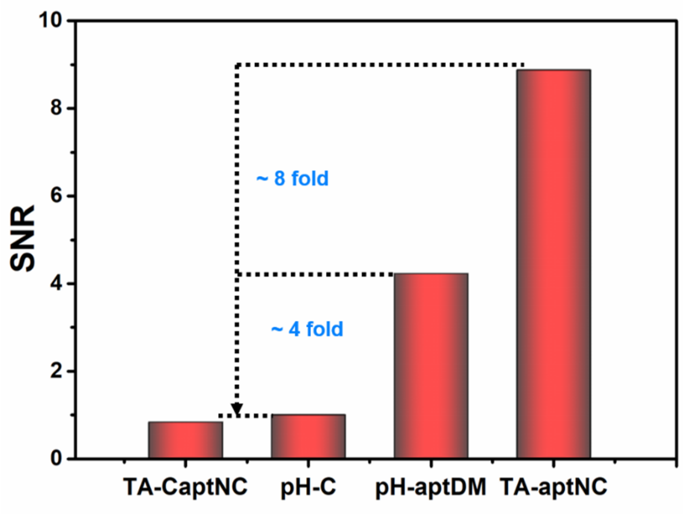

Figure S7. The corresponding histogram of the fluorescence intensity ratios of different probes, including TA-CaptNC (the mixture of CDM_{1I}, CDM_{2I} and CDM_{3I}), pH-C group (the mixture of DM_{1C}, DM_{2C} and DM_{3C}), pH-aptDM (the corresponding monovalent probe) and TA-aptNC (the mixture of DM_{1I}, DM_{2I} and DM_{3I}) treated with SMMC-7721 cells at pH 6.3 to that at pH 7.4 in Fig. 2B.

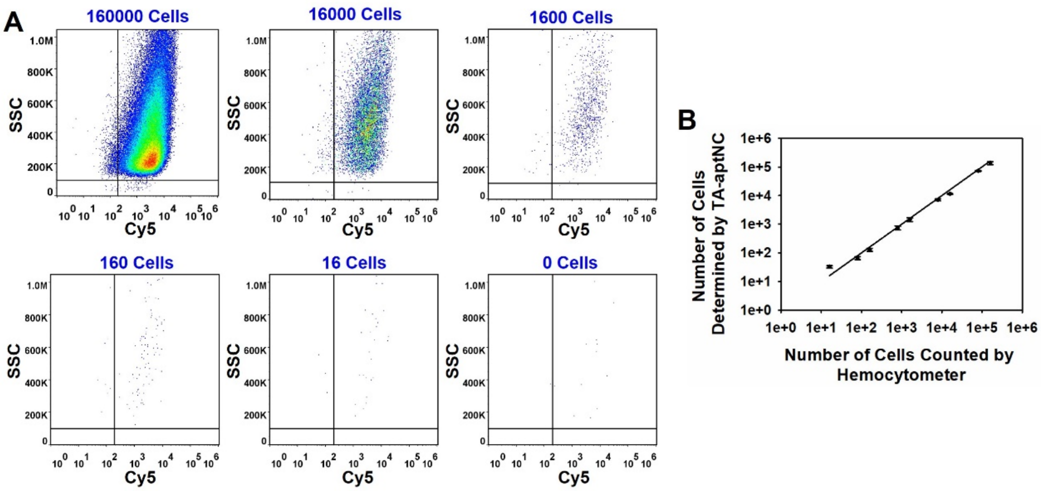

Figure S8. Quantitative detection of SMMC-7721 cells by TA-aptNC-based *in situ* assembly multivalent nanodevice. (A) Flow cytometry analysis of SMMC-7721 cells with decreasing cell amounts in 200 μL binding buffer using the proposed *in situ* assembled muitivalent nanosystem. (B) Calibration curve illustrating the relationship between the number of SMMC-7721 cells counted by hemocytometer and the amount of SMMC-7721 cells detected by TA-aptNC. Error bars show the standard deviation of three repetitive experiments.

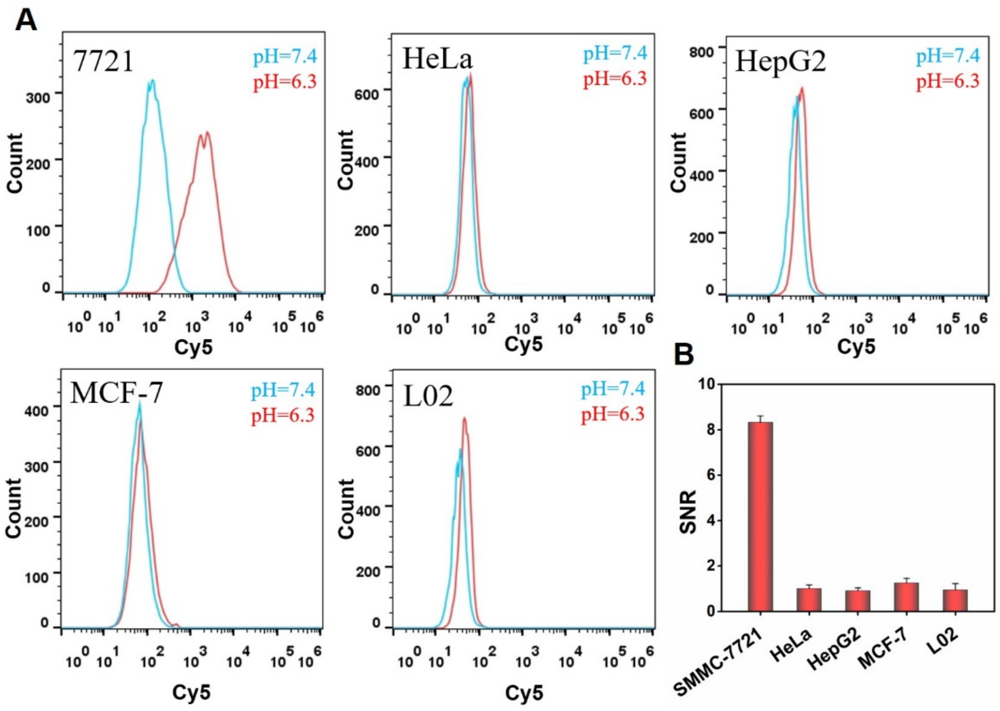

Figure S9. Specificity investigation of the proposed method. (A) Flow cytometric assays of various cells (SMMC-7721, HeLa, HepG2, MCF-7 and L02 cells) after incubation with the mixture of DM_{1I}, DM_{2I} and DM_{3I} (TA-aptNC) in binding buffer at different pH values. (B) The corresponding fluorescence intensity ratios SNR of the probes at pH 6.3 to that at pH 7.4 for different cells in (A). Error bars show the standard deviation of three repetitive experiments.

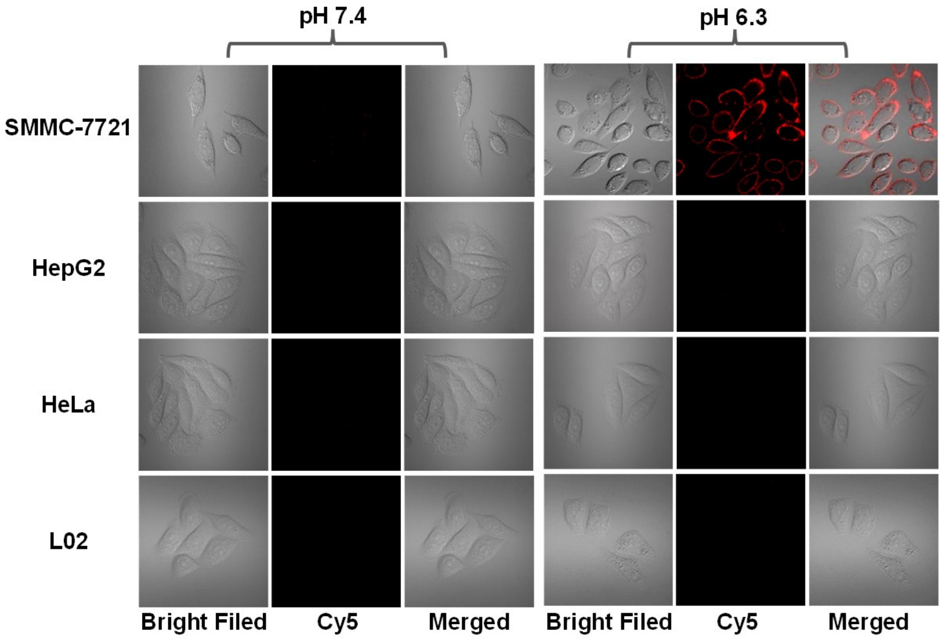

Figure S10. Specificity investigation of the TA-aptNC-based *in situ* strategy by confocal microscopy after incubation with different cells (SMMC-7721, HepG2, HeLa and L02 cells) in binding buffer with different pH values at 37 °C for 1 h (scale bar: 20 μm).

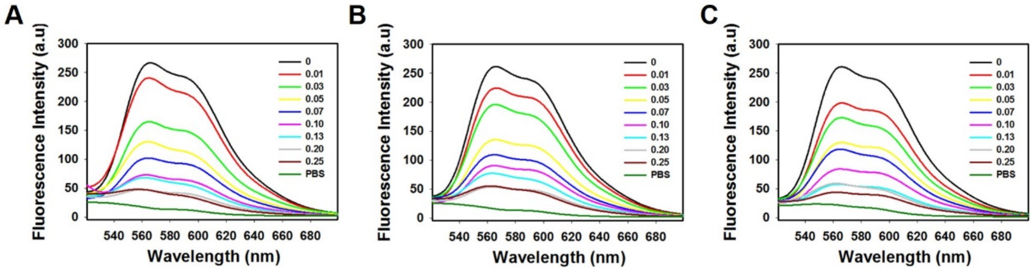

Figure S11. Drug payload investigation of three DNA modules. Fluorescence spectra of Dox (1 μM) with increasing molar ratios of (A) DM_{1I} or (B) DM_{2I} or (C) DM_{3I} to Dox. The fluorescence quenching implies drug loading into DNA modules.

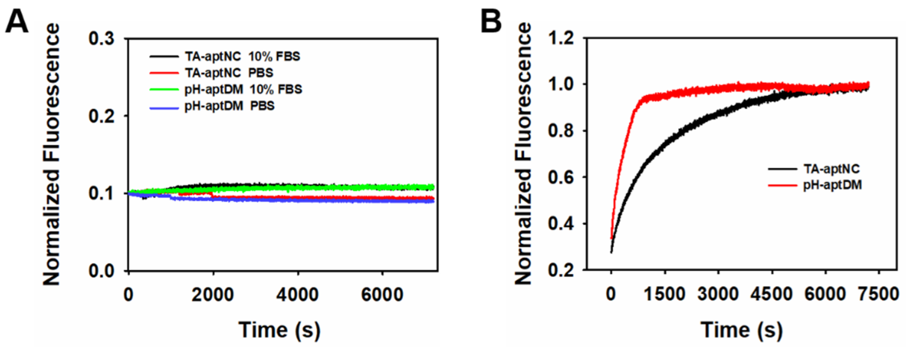

Figure S12. Characterization of drug release. Time-lapse fluorescence monitoring of Dox diffused from pH-aptDM or TA-aptNC at 37 °C in PBS or 10% fetal bovine serum (A) and in PBS buffer containing 1.5 U/mL DNase I at 37 °C (B).

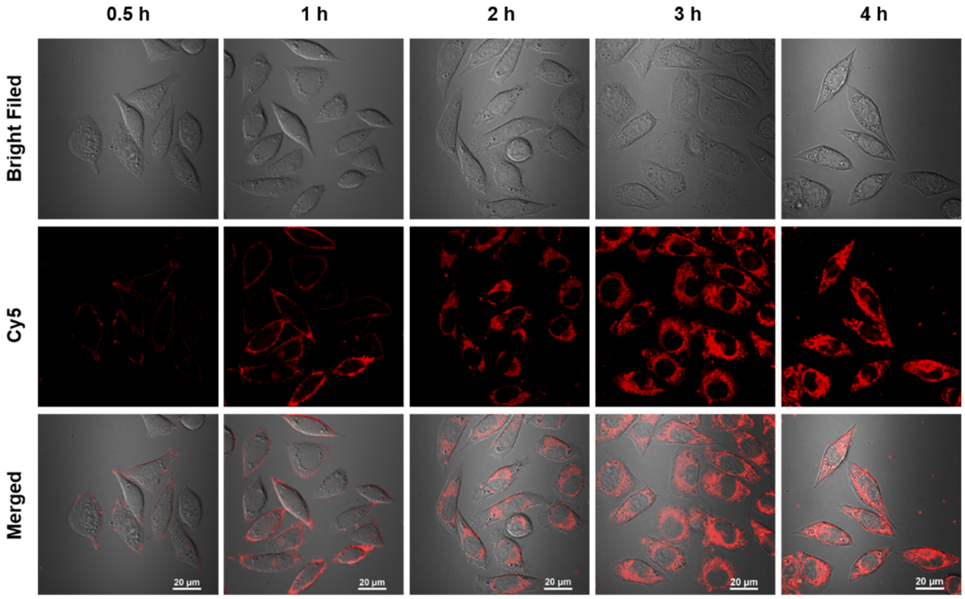

Figure S13. Time-dependent internalization of the mixture of three DNA modules in SMMC-7721 cells revealed by confocal microscopy. SMMC-7721 cells were incubated with the mixture of three DNA modules at 37 °C for 0.5, 1, 2, 3 and 4 h, respectively. (Scale bar: 20 μm).

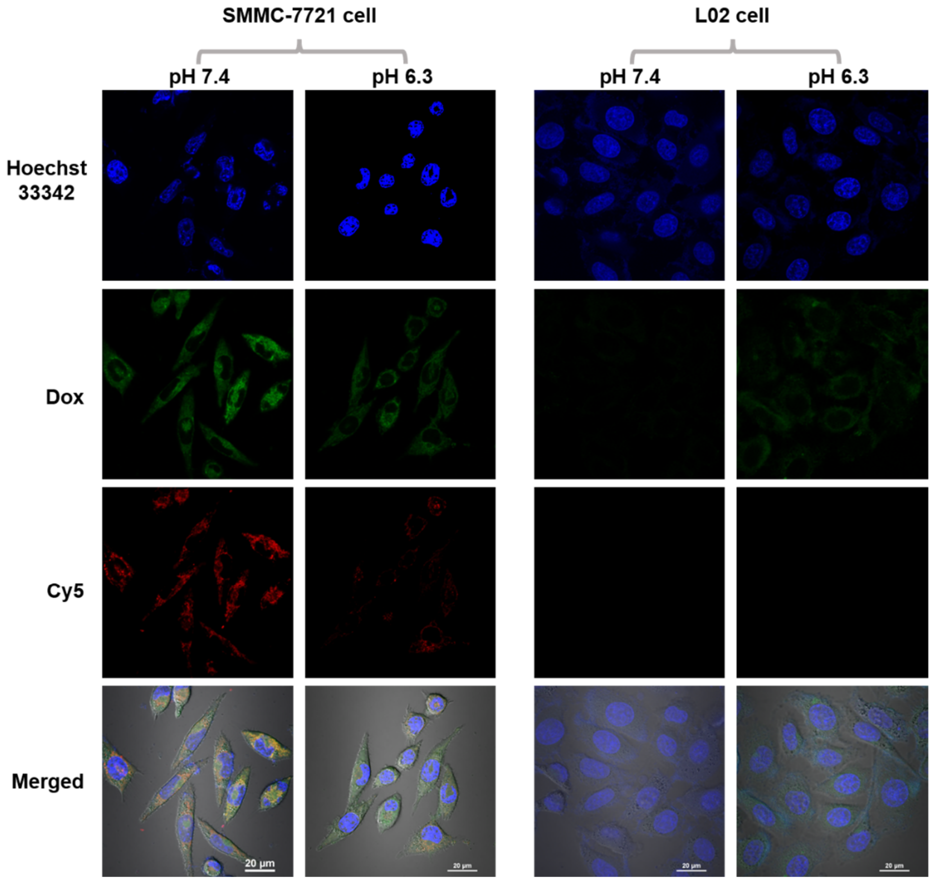

Figure 14. Selective drug transport of the mixture of three Dox-loaded pH-aptDMs (pH-aptDMs-Dox) against targeted SMMC-7721 cells. Target cancer cells and normal L02 cells were incubated with the mixture (1 μM Dox equivalents) in a simulated solution (pH 6.3 or 7.4) at 37 °C for 3 h. Scale bar: 20 μm.

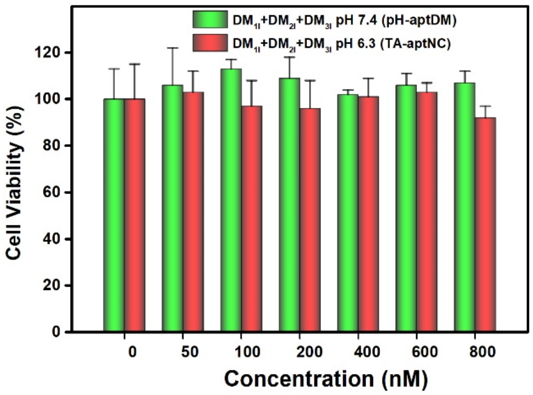

Figure S15. MTS assay of the mixture of three DNA modules to SMMC-7721 cells at different pH values. The data error bars indicate mean ± SD (n = 3).

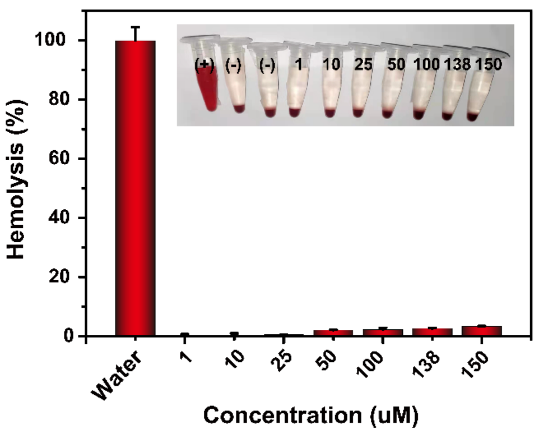

Figure S16. Hemolysis assay of erythrocytes at various concentrations of the mixture of three pH-aptDMs. Inset: photographs of hemolysis of RBCs treated with the mixture at different concentrations ranging from 1 to 150 μM for 12 h. The presence of red hemoglobin in the supernatant indicated damaged erythrocytes. Samples of RBCs incubated with water (+) and PBS (-) was used as positive and negative control, respectively.

References

1 Li J, Xun KY, Zheng LY, Peng XY, Qiu LP, Tan WH. DNA-based dynamic mimicry of membrane proteins for programming adaptive cellular interactions. J Am Chem Soc. 2021; 143: 4585-4592.

2 Li WS, Yang XH, He LL, Wang KM, Wang Q, Huang J, et al. Self-assembled DNA nanocentipede as multivalent drug carrier for targeted delivery. ACS Appl Mater Interfaces. 2016; 8: 25733-25740.

3 Kaiser CE, Van Ert NA, Agrawal P, Chawla R, Yang DZ, Hurley LH. Insight into the complexity of the i-Motif and G-Quadruplex DNA structures formed in the KRAS promoter and subsequent drug-induced gene repression. J Am Chem Soc. 2017; 139: 8522-8536.
